# Five-year results of the complete versus culprit vessel percutaneous coronary intervention in multivessel disease using drug-eluting stents II (CORRECT II) study: a prospective, randomised controlled trial

**DOI:** 10.1007/s12471-019-1252-3

**Published:** 2019-03-13

**Authors:** N. D. Fagel, F. C. van Nooijen, M. Maarse, T. Slagboom, J. P. Herrman, R. J. van der Schaaf, G. Amoroso, M. S. Patterson, G. J. Laarman, M. J. Suttorp, M. A. Vink

**Affiliations:** 1 Department of Cardiology, OLVG Hospital, Amsterdam, The Netherlands; 2 Department of Cardiology, Waterland Hospital, Purmerend, The Netherlands; 3 Department of Cardiology, Elisabeth-TweeSteden Hospital, Tilburg, The Netherlands; 4 Department of Cardiology, Sint Antonius Hospital, Nieuwegein, The Netherlands

**Keywords:** Acute coronary syndrome, Percutaneous coronary intervention, Coronary artery disease, Multivessel disease, Drug-eluting stent, Treatment strategy

## Abstract

**Objectives/background:**

In patients with multivessel coronary artery disease (MVD) the decision whether to treat a single culprit vessel or to perform multivessel revascularisation may be challenging. The purpose of this study was to evaluate the long-term outcome of multivessel percutaneous coronary intervention (MV-PCI) versus culprit vessel only (CV-PCI) in patients with stable coronary artery disease or non-ST elevation acute coronary syndrome.

**Methods:**

In this dual-centre, prospective, randomised study a total 215 patients with MVD were randomly assigned to MV-PCI or CV-PCI. The primary endpoint was the occurrence of major adverse cardiac events (MACE) including death, myocardial infarction (MI), and repeat revascularisation. Secondary endpoints were the combined endpoint of death or MI, the individual components of the primary endpoint, and the occurrence of stent thrombosis. Patients were followed up to 5 years after enrolment.

**Results:**

The occurrence of the primary endpoint was similar at 28% versus 31% in the MV-PCI and CV-PCI group, respectively (hazard ratio [HR] 0.87, 95% confidence interval [CI]: 0.53–1.44, *p* = 0.59). The rate of repeat revascularisation was 15% versus 24% (HR 0.59, 95% CI 0.32 to 1.11, *p* = 0.11), whereas definite or probable stent thrombosis occurred in 2% versus 0% (*p* = 0.44).

**Conclusions:**

In this randomised study comparing the strategies for MV-PCI and CV-PCI in patients with MVD, no difference was found in the occurrence of MACE after 5 years. We observed a numerically higher rate of death or MI and a lower rate of repeat revascularisation after MV-PCI, although these findings were not statistically significant.

## What’s new?

- Addressing, in a randomised fashion, the problem regarding the decision to perform single or multivessel percutaneous coronary intervention (PCI) in patients with stable coronary artery disease (SCAD) or non-ST elevation acute coronary syndrome (NSTE-ACS) in cases of multivessel disease.
- Adding to the above discussion in a hypothesis-generating way.
- Single (culprit) vessel PCI might be non-inferior to multivessel PCI for patients with SCAD/NSTE-ACS with respect to clinical endpoints and major adverse cardiac events.

## Introduction

Percutaneous coronary intervention (PCI) provides symptom relief and/or improves the clinical outcome for patients with obstructive coronary artery disease that has resulted in stable angina pectoris or acute coronary syndrome (ACS) [1–3]. In patients with multivessel coronary artery disease (MVD) the choice whether to treat a single culprit vessel or to perform multivessel revascularisation is often a matter of debate. Available data on the comparison between these treatment strategies are largely observational and difficult to interpret [4, 5]. Current clinical practice guidelines do not give a clear recommendation as to which strategy is preferred [2, 3, 6]. For that reason, the decision whether to perform PCI of the culprit vessel only (CV-PCI) or to pursue complete revascularisation (MV-PCI) is made largely on an individual basis, taking into account available patient data (non-invasive ischaemia testing, coronary angiography, echocardiographic findings and/or electrocardiographic changes).

The CORRECT I study was one of the first randomised, controlled trials to compare MV-PCI with CV-PCI in patients with stable coronary artery disease (SCAD) or non-ST elevation acute coronary syndrome (NSTE-ACS). In this study using bare-metal stents (BMS) the MV-PCI strategy had a lower procedural success rate and a similar incidence of major adverse cardiac events (MACE) up to 1 year of follow-up, as compared with CV-PCI [7]. Obviously, patients treated for MVD would benefit from treatment with drug-eluting stents (DES) since less in-stent restenosis is seen with these types of stents as compared with BMS [8, 9]. However, the potential long-term hazards of implanting multiple stents as well as the fact that prognosis may not be enhanced by MV-PCI underline the need to address this matter in a randomised fashion. Therefore, the CORRECT II study using DES was designed, in which CV-PCI was compared with MV-PCI in patients with MVD and an indication for revascularisation in the context of SCAD or NSTE-ACS.

## Methods

### Study design

The trial was performed in two centres in The Netherlands (OLVG, Amsterdam and Sint Antonius Ziekenhuis, Nieuwegein). The study was designed in 2005 as a prospective, open-label, randomised, controlled trial. Patient recruitment was from 2005 to 2011. Patients were recruited among all patients admitted to the hospital in whom coronary angiography was performed due to SCAD or NSTE-ACS. All patients with significant coronary lesions (≥70%) in ≥2 coronary vessels with a diameter ≥2 mm and length of ≤30 mm were eligible for participation. The culprit vessel was identified by two independent interventional cardiologists on the basis of clinical information, including electrocardiography (17.0%), echocardiography (4.8%), scintigraphy (6.3%) or coronary angiography (89.4%). Patients with ST-elevation myocardial infarction were excluded. Other major exclusion criteria were co-morbidity limiting the life expectancy to <1 year, stenosis of a venous or arterial bypass graft, participation in another clinical trial or living abroad. Previous coronary artery bypass graft with a treatable stenosis in a native coronary artery was allowed. Eligible patients were randomly assigned to either MV-PCI or CV-PCI. The study complied with the principles set out in the Declaration of Helsinki. Written informed consent was obtained from each patient.

### Procedure

All procedures were performed according to the study protocol using DES. Invasive evaluation by fractional flow reserve (FFR), intravascular ultrasound or optical coherence tomography was not used in any of the patients since this was not yet common practice at the time this study was carried out. If a staged procedure was carried out, a clear explanation was given. In this case PCI had to be performed within 14 days. Strategy success was defined as the achievement of Thrombolysis in Myocardial Infarction (TIMI) grade III flow after treatment of the culprit lesion or all significant lesions, depending on strategy assignment, and an angiographic residual stenosis <30% of the treated lesion(s).

### Follow-up

Total duration of clinical follow-up was 5 years. Follow-up was performed by telephone interview each year. MACE were determined at these follow-up telephone interviews or if the patient was admitted to the hospital. If MACE occurred, information was requested from the treating hospital. Use of medication, Canadian Cardiovascular Society grade of angina pectoris and re-hospitalisation were determined at each follow-up visit. Vital status was obtained from local authorities if a patient could not be reached.

### Outcome

The primary endpoint was the occurrence of MACE comprising death, myocardial infarction (MI), and repeat revascularisation. Secondary endpoints were the combined endpoint of death or MI, the individual components of the primary endpoint, the occurrence of stent thrombosis, and angina class. The diagnosis of new MI was based on new >0.03 s Q waves on electrocardiography or a rise in creatine kinase enzyme above 200 U/l or in its MB fraction above 20 U/l.

### Statistical analysis

It was calculated that 462 patients were needed to detect a minimum treatment difference of 12% in the primary endpoint with a power of 80%, and a two-sided significance level set at 0.05, taking a 10% loss to follow-up into account. Assumptions were made largely based on the results of the CORRECT I study [7]. Data are presented as mean (±SD) for normally distributed, continuous variables and as frequencies (percentages) for categorical variables. Differences at baseline were tested with Student *t* test or the Wilcoxon rank-sum test for continuous variables and chi-square test for categorical variables. Tests were 2‑tailed and a value of *p* ≤ 0.05 was considered statistically significant. Endpoints were analysed according to the intention-to-treat principle. The cumulative incidence rate of the primary and secondary clinical endpoints was estimated with the Kaplan-Meier method. We calculated hazard ratios (HR) with 95% confidence intervals (CI) with the Cox-proportional hazard model. Treatment allocation was the only variable. Follow-up was censored at 5 years. Differences in event rates were then tested for significance by the log-rank test. Statistical analysis was done with SPSS (version 22.0 for Windows, SPSS, Inc., Chicago, IL, USA).

## Results

**Fig. 1:**
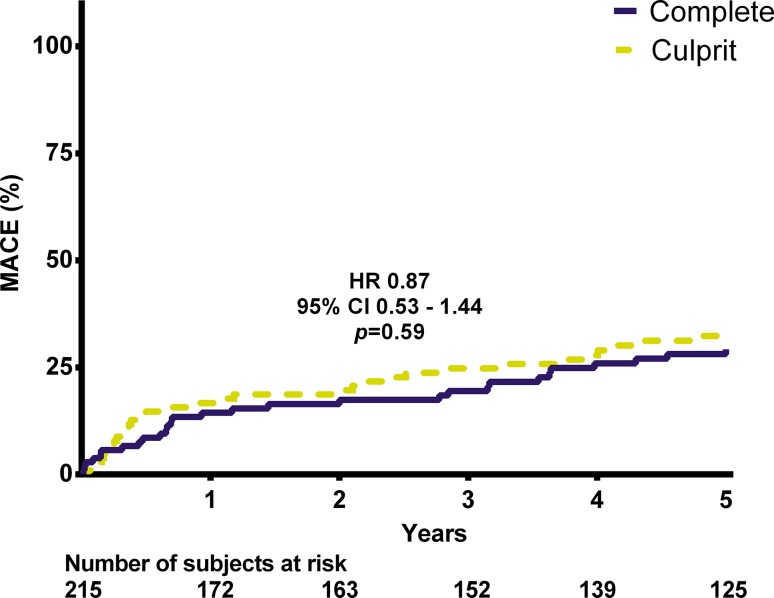
Kaplan-Meier curve for the incidence of major adverse cardiac events (*MACE*) at 5‑year follow-up. (*HR* hazard ratio, *CI* confidence interval)

**Fig. 2:**
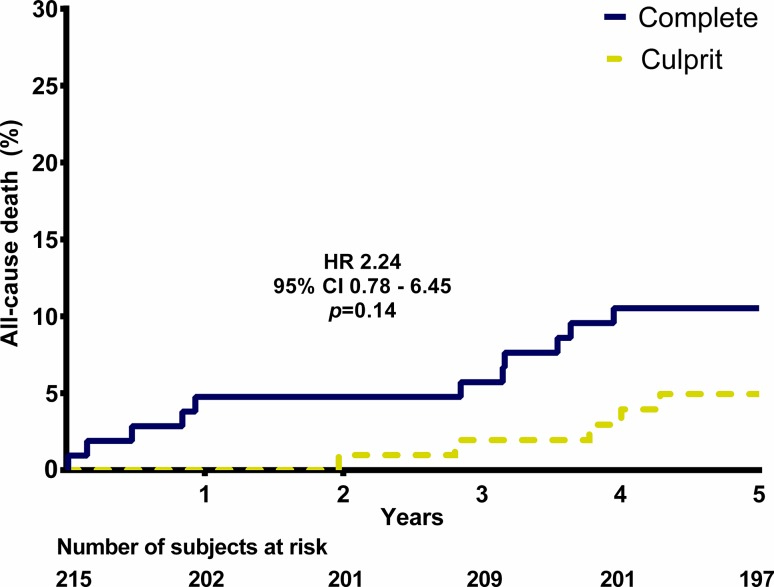
All-cause death. (*HR* hazard ratio, *CI* confidence interval)

**Fig. 3:**
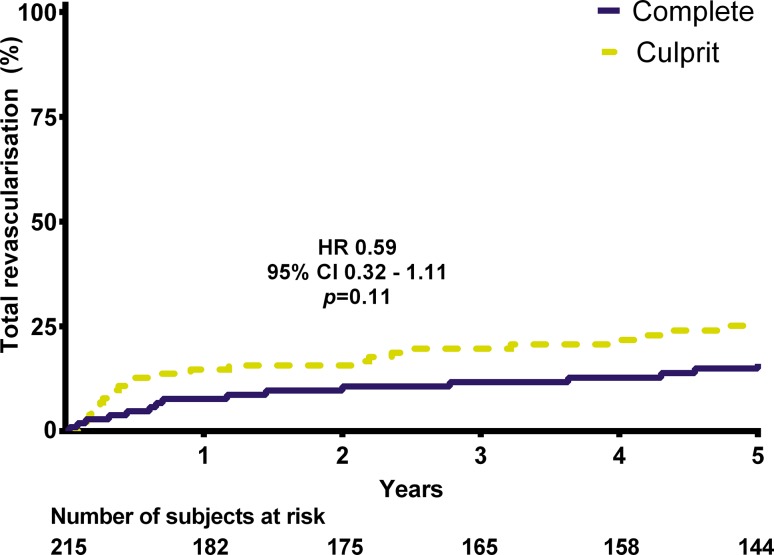
Total revascularisation. (*HR* hazard ratio, *CI* confidence interval)

**Fig. 4:**
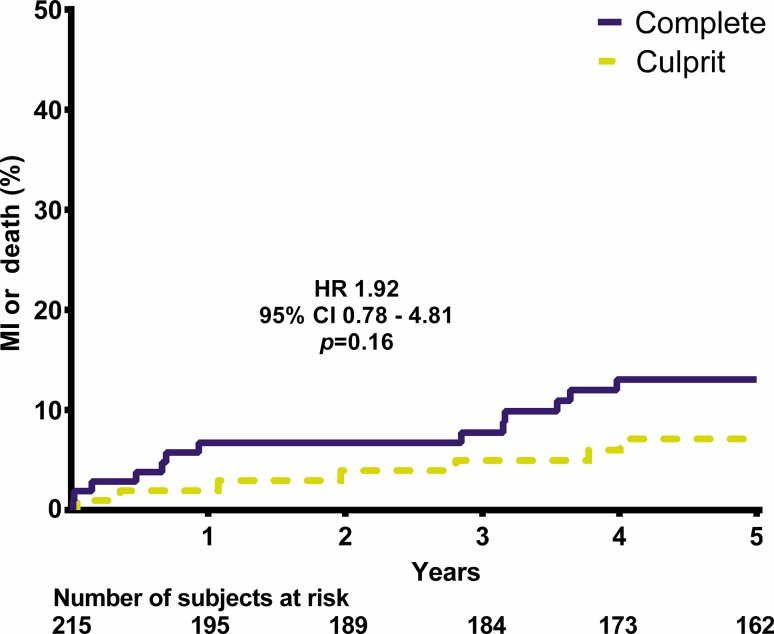
Myocardial infarction (*MI*) or death. (*HR* hazard ratio, *CI* confidence interval)

A total of 215 patients were included in the study. The baseline clinical characteristics are shown in Tab. 1 and were well matched. There was no difference in angiographic and procedural characteristics at baseline (Tab. 2). Strategy success rate was 94 and 96% for MV-PCI and CV-PCI, respectively (*p* = 0.75). Patients mainly had two-vessel disease (94% both groups). At 5‑year follow-up vital status was available for 94% of all patients. The occurrence of the primary endpoint was similar in the MV-PCI and CV-PCI groups at 28% versus 31% (HR 0.87, 95% CI: 0.53–1.44, *p* = 0.59); see Tab. 3. Fig. 1 shows the Kaplan-Meier curve for the incidence of MACE at 5‑year follow-up. With 11 cases (10%) in the MV-PCI group and 5 cases (5%) in the CV-PCI group, there was a numerically small difference in all-cause death, which was not statistically significant (HR 2.24, 95% CI: 0.78–6.45, *p* = 0.14) (Fig. 2). A small but non-significant difference was found for repeat PCI rate with fewer procedures in the MV-PCI than in the CV-PCI group (13% vs 20%, HR 0.65, 95% CI: 0.33–1.27, *p* = 0.20). If re-PCI or coronary artery bypass graft was combined there was no difference between the study groups, as is shown in Fig. 3 (15% vs 24%, HR 0.59, 95% CI: 0.32–1.11, *p* = 0.11). The occurrence of MI at 5‑year follow up was similar for the MV-PCI and CV-PCI group, with a rate of 4% versus 3% (HR 1.36, 95% CI: 0.30–6.08, *p* = 0.69). When death or MI were combined, the rate in both groups was similar as well (12% vs 7%, HR 1.92, 95% CI: 0.78–4.81, *p* = 0.16) (Fig. 4). The incidence of definite or probable stent thrombosis was low in both MV-PCI and CV-PCI groups at a rate of 2% versus 0% (*p* = 0.44).

**Table 1:** Baseline characteristics

baseline characteristic	culprit (*n* = 103)	complete (*n* = 105)	*p*-value
age (years), mean (SD)	64.4 (10)	66.4 (10)	0.138
male, *n* (%)	78 (76)	72 (69)	0.281
female, *n* (%)	25 (24)	33 (31)	0.281
previous MI, *n* (%)	35 (34)	36 (34)	1.000
CABG, *n* (%)	1 (1)	1 (1)	1.000
PCI, *n* (%)	14 (14)	21 (20)	0.267
hypertension, *n* (%)	55 (53)	52 (50)	0.582
current smoker, *n* (%)	18 (19)	25 (25)	0.427
diabetic, *n* (%)	17 (17)	25 (24)	0.228
hypercholesterolaemia, *n* (%)	33 (32)	31 (30)	0.765
anticoagulation, *n* (%)	7 (7)	5 (5)	0.567
anti-platelet agent, *n* (%)	96 (93)	98 (93)	1.000
nitrate, *n* (%)	38 (37)	38 (36)	1.000
beta-blocker, *n* (%)	90 (88)	90 (86)	0.681
calcium antagonist, *n* (%)	26 (25)	37 (35)	0.133
diuretics, *n* (%)	20 (20)	24 (23)	0.612
ACE inhibitor, *n* (%)	28 (27)	37 (35)	0.233
angiotensin II receptor blocker, *n* (%)	15 (15)	9 (9)	0.198
statin, *n* (%)	96 (93)	93 (89)	0.336
*angina pectoris CCS*, *n* (%)			
– I	2 (2)	4 (4)	
– II	36 (35)	33 (31)	
– III	30 (29)	24 (23)	
NSTE-ACS	34 (33)	42 (40)	0.316

*MI* myocardial infarction, *CABG* coronary artery bypass graft, *PCI* percutaneous coronary intervention, *CCS* Canadian Cardiovascular Society grade,* NSTE-ACS* non-ST elevation acute coronary syndrome

**Table 2:** Procedural characteristics

procedural characteristic	culprit (*n* = 103)	complete (*n* = 105)	*p*-value
number of lesions ≥70%, mean (SD)	2.1 (0.2)	2.1 (0.23)	0.973
*significantly diseased vessels*, *n* (%)			
– 2-vessel disease	97 (94)	99 (94)	
– 3-vessel disease	6 (6)	6 (6)	1.000
*location of culprit vessel*, *n* (%)			
– LAD	44 (43)	48 (46)	
– RCX	29 (28)	22 (21)	
– RCA	30 (29)	35 (33)	0.472
*location of non-culprit vessel lesion 1*, *n* (%)			
– LAD	36 (35)	29 (28)	
– RCX	35 (34)	49 (47)	
– RCA	32 (31)	27 (26)	0.174
strategy success	99 (96)	99 (94)	0.748
*pre-procedural TIMI grade flow* *Culprit vessel*			
– 0	6 (6)	4 (4)	0.508
– I	6 (6)	3 (3)	
– II	7 (7)	11 (11)	
– III	83 (81)	86 (83)	
*type of stent*			
– taxus	59	55	
– xience	13	18	
– pro-Kinetic	0	1	
– promus	13	13	
– janus	1	3	
– cypher	3	0	
– titan	1	0	
– combination	2	4	
– no stent	0	1	
*peri-procedural complications*, *n* (%)			
– side branch occlusion	3 (3)	5 (1)	0.722
– coronary spasm	1 (1)	0 (0)	0.490
– coronary embolism	0 (0)	0 (0)	
– acute stent thrombosis	0	0	
– bleeding	0	0	
– MI	1 (1)	4 (4)	
– TIA/CVA	0	0	
– death	0	0	0.370
– other	4 (4)	8 (8)	0.374

*LAD* left anterior descending artery, *RCX* ramus circumflex artery, *RCA* right coronary artery, *TIMI* thrombolysis in myocardial infarction, *MI* myocardial infarction, *TIA* transient ischaemic attack, *CVA* cerebrovascular accident

**Table 3:** Clinical events at 5‑year follow-up

clinical events	complete *n* = 105	culprit *n* = 103	HR	CI (95%)	*p*-value
overall MACE (%)	29 (28)	32 (31)	0.87	0.53–1.44	0.589
all-cause mortality	11 (10)	5 (5)	2.24	0.78–6.45	0.135
acute MI	4 (4)	3 (3)	1.36	0.30–6.08	0.688
CABG	2 (2)	4 (4)	0.51	0.09–2.77	0.432
repeat PCI	14 (13)	21 (20)	0.65	0.33–1.27	0.204
total revascularisation (PCI or CABG)	16 (15)	25 (24)	0.59	0.32–1.11	0.105
combined death or MI	13 (12)	7 (7)	1.92	0.78–4.81	0.164
*angina pectoris at 5‑year FU*					
– class I	3 (3)	3 (3)			
– class II	9 (9)	8 (8)			
– class III	2 (2)	0 (0)			
*stent thrombosis*					0.442
– definite	1 (1)	0 (0)			
– probable	1 (1)	0 (0)			

*HR* hazard ratio, *CI* confidence interval, *MACE* major adverse cardiac events, *MI* myocardial infarction, *CABG* coronary artery bypass graft, *PCI* percutaneous coronary intervention, *FU* follow-up

In total 61.4% of the included patients had stable angina and 35.3% had an ACS. We found a higher MACE rate in the patients presenting with ACS, but no significant difference when the randomisation groups are compared: 16 MV-PCI versus 12 CV-PCI (38% vs 35%, HR 1.11, 95% CI: 0.52–2.35, *p* = 0.78). Furthermore, 33 of all observed events (54% of total MACE) occurred in patients presenting with SCAD. The event rate in the groups was comparable: 13 MV-PCI versus 20 CV-PCI (21% vs 29%, HR 0.68, 95% CI 0.34–1.37; *p* = 0.285).

## Discussion

In the present study comparing MV-PCI and CV-PCI treatment strategies in patients with MVD, we found no significant difference in the occurrence of the combined endpoint of death, MI, or repeat revascularisation up to 5 years after enrolment. In patients with MVD complete percutaneous revascularisation could potentially have adverse effects both in the short and long term, varying from contrast-induced nephropathy, procedural complications, and a higher risk of stent-related events (restenosis, neo-atherosclerosis or stent thrombosis). From observational data, it is acknowledged that a considerable amount of cardiac events in patients with known coronary artery disease are caused by early, late or very late stent thrombosis, which entail a higher mortality risk compared to MI not related to a stented site [10]. These results suggest that treating different lesions and implanting more stents at the index procedure might be of potential harm in the longer term.

Results of the COURAGE trial showed no long-term survival benefit of PCI on top of optimal medical therapy for patients with SCAD [11]. Moreover, the recently published ORBITA trial even raised questions about symptom relief in patients with stable angina undergoing PCI, when compared to a sham procedure [12]. Although the interpretation of the results of this latter trial is subject to debate, the long-term hazards of stenting may support a more restrictive approach even in multivessel disease.

Literature addressing the dilemma of MV-PCI versus CV-PCI in patients with NSTE-ACS or SCAD is scarce. Most studies are observational and have several limitations. A sub-analysis of the ACUITY trial suggests that incomplete revascularisation at the index procedure in patients with ACS is associated with a higher 1‑year event rate [13]. In a recently published meta-analysis the results of 12 observational studies comparing MV-PCI versus CV-PCI in patients with ACS were analysed. No significant difference in mortality, repeat revascularisation, or the combined incidence of death, MI or revascularisation was found. However, the study design of the included trials was heterogeneous and there was evidence of publication bias [4]. On the other hand, another meta-analysis of nine studies including patients with NSTE-ACS or SCAD and proven MVD showed significantly lower mortality and a lower rate of non-fatal MI after complete revascularisation, when compared to incomplete revascularisation, at long-term follow-up. Surprisingly, no difference in repeat PCI was found [14]. Although the aforementioned studies have strong limitations, mainly due to study heterogeneity and publication bias, the suggestion is made that in general MV-PCI is non-inferior to CV-PCI. These observations are comparable to the results obtained in the present randomised trial.

The current study has several limitations. First, our study was terminated early due to slow patient recruitment. Therefore, it was underpowered to detect a difference in clinical endpoints. Second, although culprit identification and judgement of lesion severity were performed by two independent interventional cardiologists, this was not quantified by objective measurement. FFR can potentially be a useful tool in culprit identification and severity of coronary stenosis. At the time of design of the trial, measurement by FFR was not yet routine practice. Furthermore, in contemporary practice interventional cardiologists state they do not perform FFR even in lesions of intermediate severity [15]. Thereby the strategies in MVD as stated in the present study still reflect everyday practice. Third, we combined the results of patients presenting with SCAD and ACS. Although the decision to perform MV-PCI or CV-PCI can be challenging in both groups of patients, one should keep in mind that both conditions are different entities with different clinical outcomes in general. Fourth, patients and care providers were not blinded for the treatment arm. Although further revascularisation had to be ischaemia driven there is still a potential bias. Finally, the risk of in-stent restenosis would potentially be higher in the complete revascularisation group, since more stents were placed. Although the current study has a longer follow-up period than other studies comparing MV-PCI and CV-PCI in ACS and SCAD patients, the possibility still exists that symptomatic in-stent restenosis will occur later, after the study follow-up period [4, 7, 16].

Because of the relatively small number of patients enrolled in this study it should be considered hypothesis generating. A larger randomised, controlled trial powered for hard clinical endpoints would be of great value in order to form a clear and well-defined recommendation in international guidelines [1].

## Conclusion

Our present study is the first randomised, controlled trial evaluating the MV-PCI versus CV-PCI strategy by using DES, in patients with MVD presenting with NSTE-ACS or SCAD. After 5 years’ follow-up we observed no significant difference in the occurrence of the combined endpoint death, new MI and/or repeat revascularisation. There was a small but statistically non-significant difference between the two groups regarding a higher rate of death or MI in the MV-PCI group, and more frequent repeat revascularisation in the CV-PCI group.
